# Enhanced and Selective Absorption of Molybdenum Nanostructured Surfaces for Concentrated Solar Energy Applications

**DOI:** 10.3390/ma15238333

**Published:** 2022-11-23

**Authors:** Antonio Santagata, Maria Lucia Pace, Alessandro Bellucci, Matteo Mastellone, Eleonora Bolli, Veronica Valentini, Stefano Orlando, Elisa Sani, Simone Failla, Diletta Sciti, Daniele Maria Trucchi

**Affiliations:** 1 CNR-ISM, FemtoLAB, U.O.S. Tito Scalo, Zona Industriale, 85050 Tito Scalo, Italy; 2 CNR-ISM, DiaTHEMA Laboratory, U.O.S. Montelibretti, Via Salaria km 29.300, 00015 Monterotondo, (Rome), Italy; 3 CNR-INO, Largo Enrico Fermi, 6, 50125 Florence, Italy; 4 CNR-ISTEC, Via Granarolo, 64, 48018 Faenza, Italy

**Keywords:** solar absorbers, nanotexturing, ultra-short laser pulses, concentrated solar power, solar selectivity, thermionic converters

## Abstract

Surfaces of commercial molybdenum (Mo) plates have been textured by fs-laser treatments with the aim to form low-cost and efficient solar absorbers and substrates for thermionic cathodes in Concentrated Solar Power conversion devices. Morphological (SEM and AFM), optical (spectrophotometry), and structural (Raman spectroscopy) properties of the samples treated at different laser fluences (from 1.8 to 14 J/cm^2^) have been characterized after the laser treatments and also following long thermal annealing for simulating the operating conditions of thermionic converters. A significant improvement of the solar absorptance and selectivity, with a maximum value of about four times higher than the pristine sample at a temperature of 800 K, has been detected for sample surfaces treated at intermediate fluences. The effects observed have been related to the light trapping capability of the laser-induced nanotexturing, whereas a low selectivity, together with a high absorptance, could be revealed when the highest laser fluence was employed due to a significant presence of oxide species. The ageing process confirms the performance improvement shown when treated samples are used as solar absorbers, even though, due to chemical modification occurring at the surface, a decrease of the solar absorptance takes place. Interestingly, the sample showing the highest quantity of oxides preserves more efficiently the laser texturing. The observation of this behaviour allows to extend the applicability of the laser treatments since, by further nanostructuring of the Mo oxides, it could be beneficial also for sensing applications.

## 1. Introduction

Concentrated Solar Power (CSP) systems are designed to convert concentrated sunlight into electricity and heat. Presently, the overall conversion efficiency of conventional CSP systems depends on the physical and chemical properties of the main elements, such as lens, mirrors, solar absorbers, heat transfer fluids, and thermodynamic heat engines [1]. One of the most critical aspects affecting the CSP operation is the need of getting novel, cheap, efficient, scalable, and easy-to-fabricate thermal solar receivers (i.e., absorbers). An ideal absorber must have a high thermal and mechanical stability (i.e., high hardness and high melting point), a high spectral selectivity in the solar spectrum range of wavelengths which means in turn high absorbance and as low as possible emittance [2,3]. Moreover, it should be resistant to oxidation when working in atmospheric conditions. Different materials and solutions have been developed in recent years, from photonic crystals [4] to plasmonic metamaterial [5], with very promising values of solar-to-thermal conversion efficiency [6]. However, most of the proposed approaches are expensive and not easily scalable.

Conversely, the use of ultra-short laser pulses for developing surface texturing with periodicity at the micro- and nano-scale is an advanced but versatile technique to be considered for the fabrication of solar absorbers [7], because it allows to modify the optical properties of a vast class of materials in order to improve their performance in this specific application. Irradiation of solid targets with linearly polarized ultrashort laser pulses can, in fact, lead to the formation of periodic surface structures, the geometrical properties of which are directly dependent on the interested material nature and the electromagnetic properties of the impinging laser beam. Such structures, defined as Laser Induced Periodic Surface Structures (LIPSS) [8], can thus be fabricated with different geometrical patterns and periodicity in order to modify and functionalize surface properties of solids (from metals [9] to wide-bandgap semiconductors [10]) without having to use aggressive and time-consuming techniques, such as chemical processing or optical and/or electronic lithography.

The physical phenomena that give rise to the formation of LIPSS are several and directly related to the nature of the irradiated materials. In the case of strongly absorbing materials, such as metals, the widely accepted theory affirms that LIPSS originated from interference between the incident laser wave and the ones scattered or excited at the material’s surface [11], such as surface plasmon polaritons [12,13]. In essence, the interference can modulate the laser beam energy intensity so that periodic ripples can be formed via localized ablation. The available and widely accepted categorization of LIPSS divide the possible structures in Low Spatial Frequency LIPSS [8], or LSFL (i.e., ripples periodicity, *Λ*, comparable or about halved the laser beam wavelength *λ*/2 ≤ *Λ* ≤ *λ*) and High Spatial Frequency LIPSS—HSFL [14] (i.e., ripples periodicity *Λ* < *λ*/2). In metals, LSFL present periodicity *Λ* ≈ *λ* [15]. Although the laser-matter mechanisms induced by ultrashort pulsed lasers can be assumed to follow non-thermal routes [16], the material’s electronic excitations involved lead towards non-equilibrium high-temperature processes where diverse compounds and phases could be obtained such as the generation at the surface of thin oxidized layers.

It was demonstrated that 1D- and 2D-LIPSS can enhance the optical absorption of materials by increasing light trapping phenomena through the augmentation of absorbing area and surface roughness [17,18] and by inducing electronically active defects in the bandgap of semiconductors [19]. The ultra-short pulsed laser technique was successfully applied on carbides [20,21], borides [22], and even transparent semiconductors, such as diamond [23] and SiC [24], to enhance the solar absorptance, however, the increase of the thermal emittance remains still a critical issue for the overall absorber performance.

The use of commercial materials is desirable to reduce costs and sintering complexity with respect to other engineered ultra-high temperature ceramics. Among commercial refractory materials, molybdenum (Mo) represents the most promising candidate for the lowest cost at this time, and above all, because of the contrary of tungsten and tantalum not being listed among the EU critical raw materials. Furthermore, Mo is also a suitable material acting as a substrate for diamond deposition or different specific coatings. In general, substrates of Mo are suitable for the development of thermionic-thermoelectric generators or hybrid high-temperature thermionic-photovoltaic devices to be used as engineered solar absorbers which can produce electricity as topping-cycle of conversion in CSP plants.

In recent years, it was demonstrated that 1D-LIPSS can be efficiently fabricated on a Mo surface by the use of femtosecond lasers to obtain a subwavelength periodicity [25]. Later, it was demonstrated by Dar et al., that LIPSS periodicity on Mo can be tuned by varying the wavelength of the impinging laser radiation [26] demonstrating that the structures induced originated from electromagnetic phenomena. Lastly, in 2022, Zhao et al., demonstrated that it is possible to reduce the reflectivity of Mo down to values averaging 15–20% in the wavelength range between 400–1000 nm [27].

This work is presented with the aim of evaluating the overall optical properties of fs-laser-treated Mo surfaces and for testing the performance of Mo-based solar absorbers in operating conditions for thermionic generators, which is at high temperature (ca. 1100 K) and in vacuum (<10^−5^ Pa). The approach followed has allowed us to detect spectral optical properties changes occurring during the operation of thermionic generators due to the produced nanostructured and nanoparticle deposited on it by four different laser fluences (i.e., 1.8, 3.6, 7.2, and 14.4 J/cm^2^) of a linearly polarized ultrashort pulsed laser source.

## 2. Materials and Methods

Laser Induced Periodic Surface Structures (LIPSSs) were fabricated on commercial molybdenum sheets (99.95% purity, thickness of 0.25 mm) by employing a Ti:Sapphire laser system able to generate linearly polarized femtosecond pulses (*λ*= 800 nm; *τ* = 100 fs; repetition rate *f* = 1 kHz). The laser beam was produced by a regeneratively amplified mode-locked “chirped-pulse” system and focused perpendicularly on the surface of the sample. Pulses were channelled through a Newport μFAB workstation(from Newport, CA, USA), fitted with a motorized nanometric resolution stage allowing accurate positioning of the sample. The diameter of the circular spot on the focal plane of the Gaussian beam profile, *w*, was evaluated to be approximately 60 µm by analysing the ablation and modification areas via SEM and by applying the method proposed by Liu [28]. In order to investigate the effect played on the optical properties by the laser treatments, the nanotexturing process was performed on macro-areas (1 × 10^−4^ m^2^) of the molybdenum samples. The samples were moved, with selectable speed, by an automated X–Y translational stage according to a raster scan (55 µm distance between two contiguous lines) along the two directions orthogonal to the laser beam propagation path. In the scanning mode, the overlapped number of pulses was adjusted by controlling the scanning speed, *v,* according to the relation *N* = *wf/v*, where *f* is the laser repetition rate fixed at 1 kHz and as already indicated, *w* is the diameter of the circular spot of the Gaussian beam profile.

For this experiment four samples, with different fabrication parameters, were surface-nano-textured. The scanning speed was fixed at 2.0 mm/s for all four laser treatments to irradiate every area of the sample with a fixed number of pulses per spot. Conversely, the single pulse fluence, *Φ_P_*, varied from 1.8 to 14.4 J/cm^2^, as it is reported in Table 1.

The microstructure was analysed by Field-Emission Scanning Electron Microscopy (FESEM, mod. SIGMA, ZEISS NTS Gmbh, Oberkochen, Germany) equipped with energy X-ray dispersive microanalysis (EDX, Model INCA energy 300; Oxford Instruments, Abingdon, UK).

The AFM images were obtained using an OmegaScope platform integrated into a LabRAM HR Evolution Raman microscope (HORIBA Ltd., Kyoto, Japan). Imaging was performed in tapping mode, setting the operational amplitude at 50 nm, and using a silicon pyramidal tip (MikroMasch HQ: NSC14/Al BS, Wetzlar, Germany) with a characteristic radius of ~8 nm, a height of ~15 µm, and 161 kHz as the resonance frequency. The scan rate was fixed at 0.5 Hz. All the AFM microscopies were acquired, filtered, and analysed using the AIST-NT SPM control software.

The optical behaviour of nanotextured Mo samples was determined at room temperature through optical transmittance and hemispherical reflectance measurements in two wavelength ranges: (1) 0.25–2.5 μm, by using a double-beam spectrophotometer (Perkin Elmer “Lambda900”, MA, USA) equipped with a 150 mm diameter Spectralon^®^-coated integration sphere; (2) 2.5–16.0 μm by means of a Fourier Transform spectrophotometer (FT-IR Bio-Rad “Excalibur”, CA, USA) equipped with a gold-coated integrating sphere and a liquid nitrogen-cooled detector. In all the cases, the spectra were acquired for a quasi-normal incidence angle.

Raman measurements were carried out using a Horiba Scientific LabRam HR Evolution confocal spectrometer (HORIBA Ltd., Kyoto, Japan) equipped with a 100 mW Oxxius laser source (λ= 532 nm), a computerized XY-table, an electron-multiplier CCD detector and an Olympus U5RE2 (Tokyo, Japan) microscope with a 100× objective (laser spot on the sample surface 0.7 μm) with a numerical aperture (NA) of 0.9, and a grating with 600 grooves/mm. All Raman spectra were recorded in backscattering geometry focalizing 10 mW at the sample and two spectra with an accumulation time of 100 s were averaged. Raman maps of 200 × 200 μm were recorded for each sample collecting nine spectra at equidistant points.

## 3. Results

We report the analysis of the main properties of the fabricated samples considering the measurements carried out before and after the thermal ageing process, which has been completed for evaluating the performance in operating conditions of the candidate samples acting as thermionic receivers.

### 3.1. Characterization of the Samples before Thermal Ageing

#### 3.1.1. Morphology and Chemical Analysis

**Figure 1:**
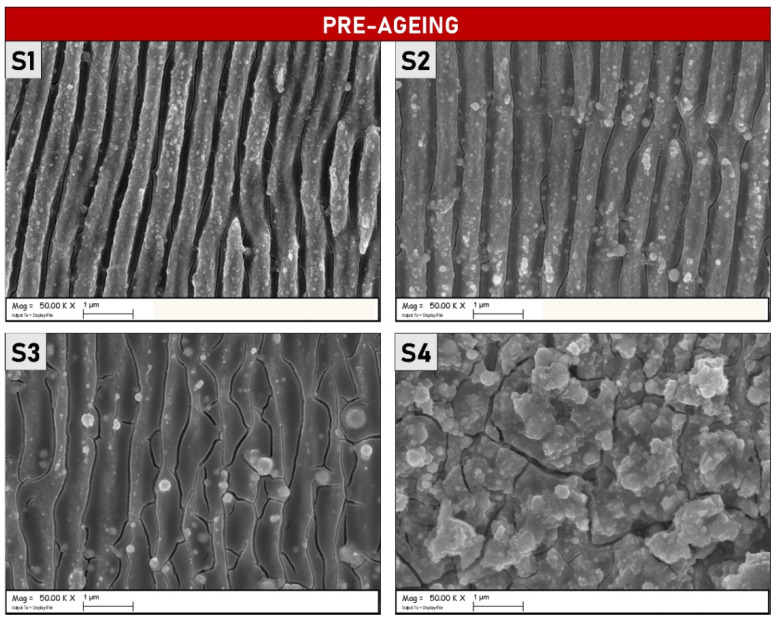
SEM micrographs (magnification 50 k×) of the four different Molybdenum samples after laser irradiation. The experimental parameters used for the (**S1**), (**S2**), (**S3**) and (**S4**) LIPSS fabrications are reported in Table 1.

**Figure 2:**
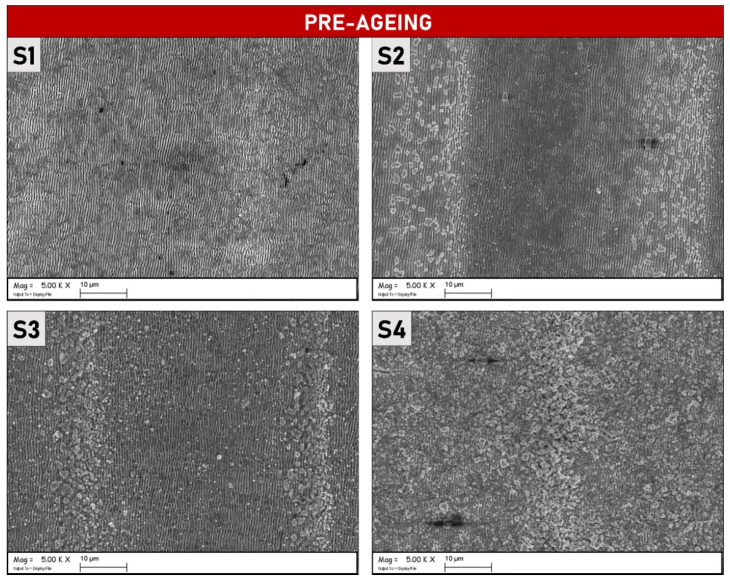
SEM micrographs (magnification 5 k×) of the four different Molybdenum samples ((**S1**), (**S2**), (**S3**) and (**S4**), experimental parameters relative to the labels are reported in Table 1) after laser irradiation.

In Figure 1 SEM micrographs (magnification 50 k×) of four different samples are reported. S1, S2, and S3 samples present regular and uniformly distributed LIPSSs running along the axis perpendicular to the laser polarization direction, in accord with the theory presented in Ref. [15], whereas S4 is completely covered by randomly distributed microstructures. At first glance the LIPSSs periodicity (*Λ* = 550 ± 60 nm, calculated using 2D-FFT) suggests, that, as expected for a molybdenum metallic material, LSFLs ripples have been generated. However, samples S2 and S3 present superficial microstructures that cover the underlying LIPSSs even if with a lesser density with respect to S4. This phenomenon is even more notable at lower magnification (see Figure 2). The microstructures on S2 and S3 are more localized in the areas where two contiguous raster lines overlap each other.

**Figure 3:**
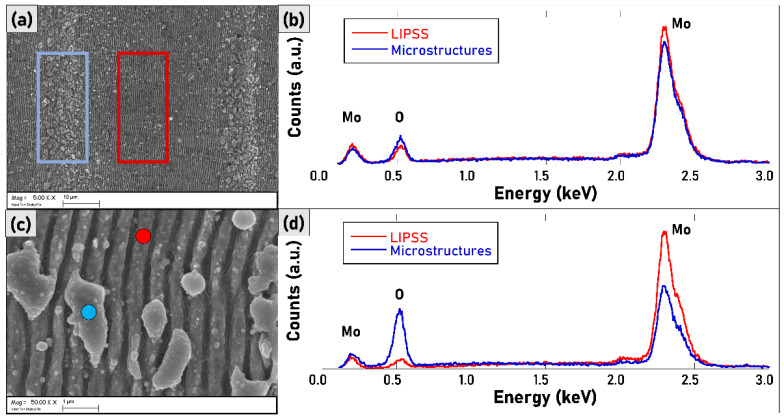
(**a**) SEM micrograph (magnification 5k×) of sample S2. The large-area regions indicated with red and blue boxes were characterized with EDX. (**b**) EDX spectra of the regions highlighted in Figure 3a. (**c**) SEM micrograph (magnification 50k×) of sample S2. The local points indicated with red and blue circles were characterized with EDX. (**d**) EDX spectra of the regions highlighted in Figure 3c.

On the other hand, the samples do not present unwanted morphological features such as craters, deep trenches, and delamination that are usually caused by irradiating the samples with an extremely high accumulated fluence. Preliminary EDX analysis confirmed that the microstructures covering the LIPSSs contain an amount of oxygen higher than the nanostructured surface (Figure 3), suggesting that the chemical composition might be a mixture of metallic and metallic oxide phases.

**Figure 4:**
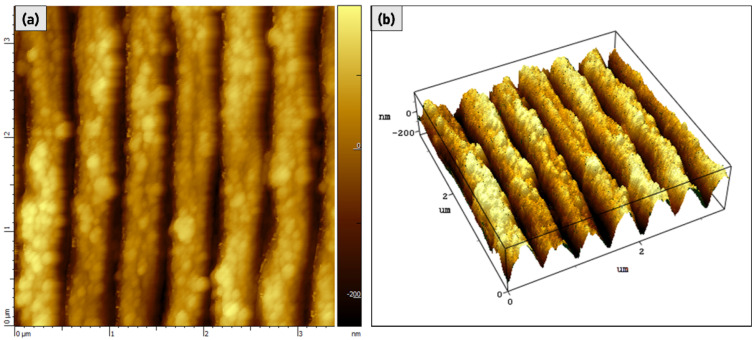
(**a**) AFM image (3.4 × 3.4 μm) of the fabricated LIPSS on S1 sample. (**b**) AFM 3D-map of the scanned area in Figure 4a.

High-resolution AFM characterization was used to provide information on the 3rd dimension, i.e., depth of the LIPSSs because of its important role played as a parameter to enhance materials’ optical absorption (Figure 4).

**Figure 5:**
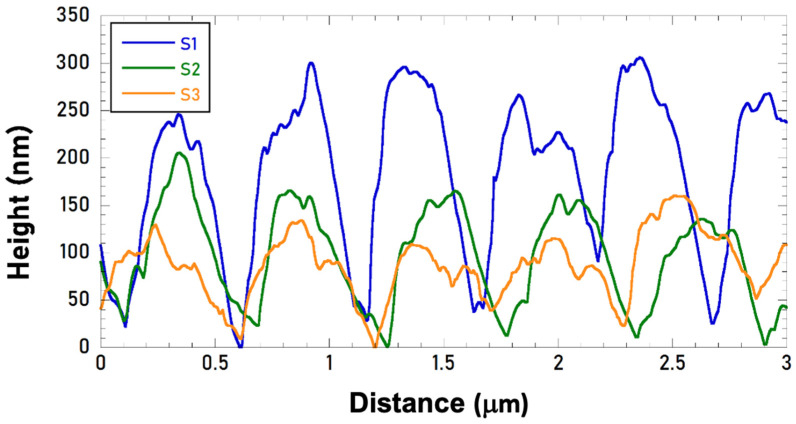
Measured surface profiles at the top of the nanostructured S1, S2, and S3 samples.

AFM characterization has demonstrated that S1 LIPSS presents a greater peak-to-trough depth, 300 ± 30 nm, than S2 and S3 samples, with a 150 ± 20 nm and 100 ± 20 nm depth, respectively. Figure 5 shows a single scanned line for S1, S2, and S3 for which the different measured depths can be easily appreciated at first sight. AFM characterizations were carried out on different areas of the samples and depth data outcomes have been assessed as an average of the measured values. We found that a higher LIPSSs depth occurs for a lower fluence. The possible reason for this behaviour can be explained either by the presence of thicker layers of oxides for samples S2 and S3 due to the higher fluences employed that fill more efficiently the valleys of the nanostructures, or even by degradation of the ripples caused by excessive laser doses. When the latter takes place, structural stress-strain between the crests and valleys of the ripples could occur by trapping the successive impinging laser beams so that periodic surface fractures, such as the ones revealed for the S3 sample, could be observed.

Since the sample S4 surface was entirely covered by randomly distributed microstructures, it was not possible to measure the depth of the underlying LIPSS.

**Figure 6:**
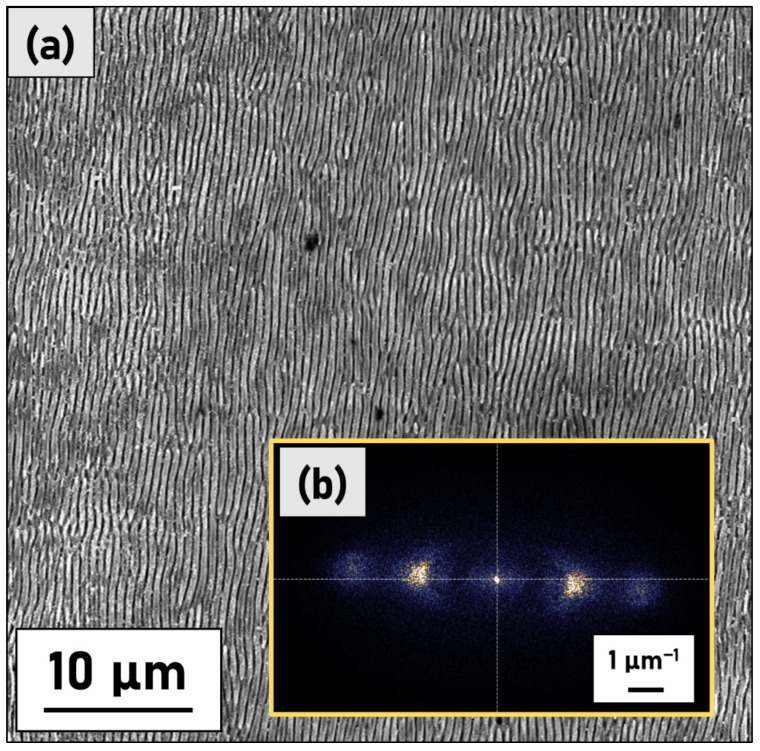
(**a**) Example of a large area SEM image of S1 used for the 2D-FFT evaluation. (**b**) In the inset is reported the 2D-FFT spectrum obtained by analysing the SEM micrograph shown in (**a**).

The software Gwyddion was used to carry out 2D Fast Fourier Transform (2D-FFT) analysis of 50 × 50 µm^2^ sized SEM images to obtain reliable values of LIPSS periodicity (Figure 6). The Fourier spectra provide two-dimensional histograms of the spatial frequencies in the original SEM images [29]. Usually, 2D-FFT images of unidimensional LIPSSs presents sickle-shaped features for which the centre position determines the most frequent spatial periods *Λ*. The “sickle” widths, on the other hand, represent the dispersion of the spatial frequencies. This allows us to quantify the spatial periodicity of the LIPSSs at 550 ± 60 nm for S1, S2, and S3 samples. It was not possible to evaluate the S4 samples FFT because the LIPSSs are mainly covered by surface oxidised microstructures.

#### 3.1.2. Optical Measurements

The optical behaviour of nanotextured molybdenum samples has been determined through spectral hemispherical reflectance *R*(*λ*) measurements. Then, the spectral absorptance *A*(*λ*) was obtained as *A*(*λ*) = 1 − *R*(*λ*).

**Figure 7:**
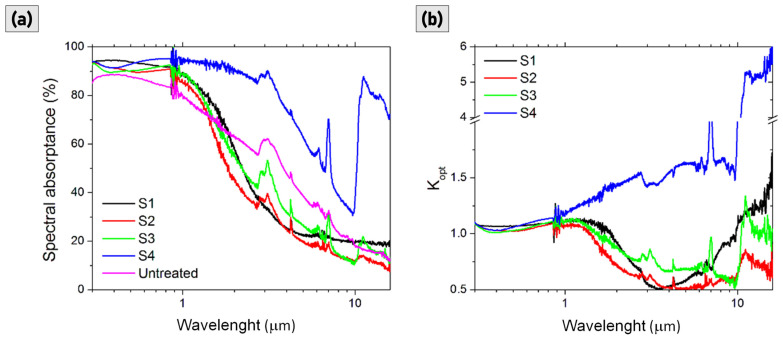
(**a**) Absorptance spectra of samples S1, S2, S3, S4, and for the untreated reference sample. (**b**) Absorptance enhancement, *K_opt_*, of samples S1, S2, S3 and S4.

Figure 7a shows the absorptance spectra of the four nanotextured samples in the wavelength range 0.3–16.0 µm, as well as the spectra measured for the reference untreated surface (pink line). Absorptance of all the treated samples has been enhanced in both visible and near-IR ranges with respect to the pristine Mo plate. For S1, S2, and S3 the absorptance increases in the wavelength range from 300 nm to about 1 µm, corresponding to the largest considerable portion of the sunlight spectral distribution. It is also notable that the absorptance of S4 remains constant up to about 3 µm, suggesting an unideal selective absorber behaviour, while the absorptance of S1, S2, and S3 drops with a steeper slope after ~1 µm, suggesting an effective enhanced behaviour for a selective solar absorber. For an easier evaluation of the effect of laser texturing on the optical properties of samples we consider, in Figure 7b, the absorptance enhancement factor *K_opt_*, defined as the ratio between the absorptance values of treated surfaces and those of the reference pristine surface [24]. Figure 7b clearly proves, in S1, S2, and S3 samples, with some differences among them, the selective enhancement of optical absorption in the UV-VIS-near-IR up to about 1 μm accompanied by the selective decreasing of absorption for longer infrared wavelengths. As for sample S4, it is completely different: the absorption enhancement is non-selective and spans, on the whole, investigated spectral range.

The reflectance response of an ideal selective solar absorber should be a step-function spanning from the minimum value of reflectance (i.e., high absorptance) up to about 1 µm (the precise cut-off depends on the operative temperature) to high reflectance (i.e., low emittance) for longer wavelengths [3]. It follows that the surface should ideally have a very low reflectance for the emitting wavelengths which, as demonstrated, is not the case for sample S4 (Figure 7a).

Aimed at evaluating the suitability of laser-nanotextured Mo plates for solar applications, the solar absorptance *α* is introduced as a comparison parameter, defined as:

(1) $$\alpha =\frac{\int_{\lambda_{1}}^{\lambda_{2}}A\left(\lambda \right)W_{solar}\left(\lambda \right)d\lambda }{\int_{\lambda_{1}}^{\lambda_{2}}W_{solar}\left(\lambda \right)d\lambda },$$

where *W_solar_*(*λ*) is the global-tilt GT 1.5 airmass and AM is the solar irradiance in the wavelength range between *λ_1_* = 300 nm and *λ_2_* = 3000 nm. It is immediate by its definition that *α* ≤ 1 (100% in percentage).

**Figure 8:**
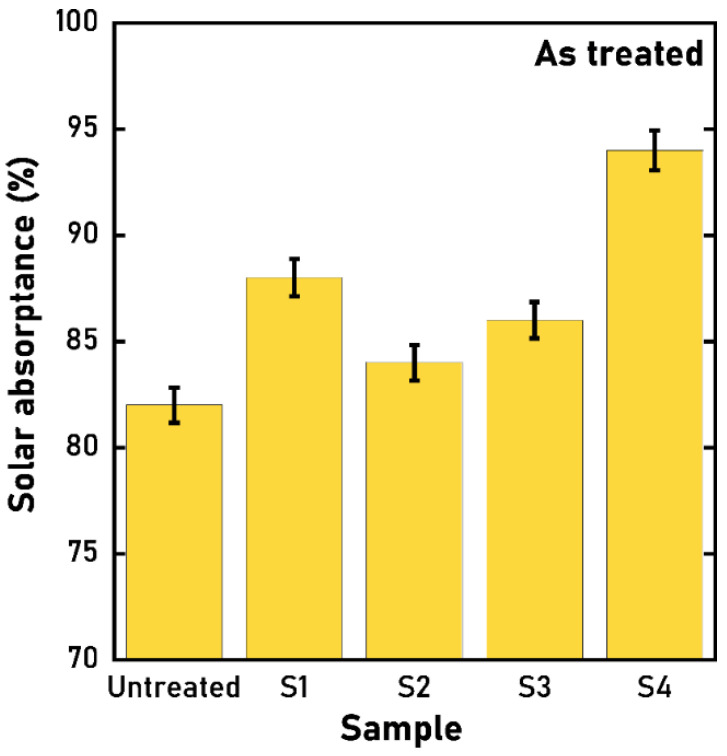
Solar absorptance of the four treated samples and the reference untreated one.

Figure 8 shows the calculated values of *α* for the treated samples. It is immediate to grasp that all the nanotextured samples have an enhanced solar absorptance although S2 and S3 present a lower *α* as their LIPSSs depth is lower. In fact, it is widely known that the increase of surface area and roughness contribute positively to the enhancement of absorptance, and S2 and S3 are deficient in this regard if compared to S1.

**Figure 9:**
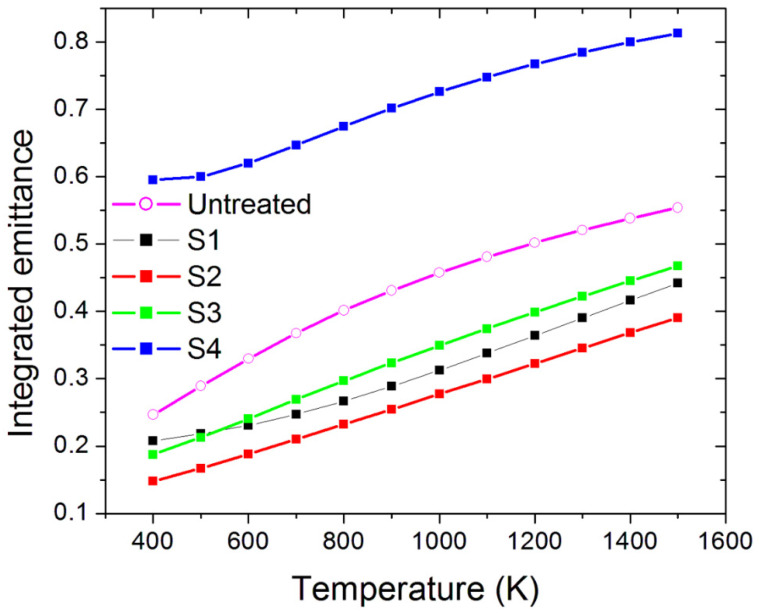
Integrated emittance of the four treated samples and the reference untreated one.

Figure 9 shows the integrated emittance (*ε*) defined as:

(2) $$\varepsilon =\frac{\int_{\lambda_{1}}^{\lambda_{3}}A\left(\lambda \right)B\left(\lambda ,\;\;T\right)d\lambda }{\int_{\lambda_{1}}^{\lambda_{3}}B\left(\lambda ,T\right)d\lambda },$$

where *B(λ,T)* is the blackbody spectral radiance at the operating absorber temperature *T*, and spectrally integrated into between *λ*_1_ = 300 nm and *λ*_3_ = 16 μm wavelength range accessible by our spectrophotometric analysis.

Sample S4 shows the highest value of emittance, which is also accompanied by the highest absorptance along the whole investigated wavelength range. A high thermal emittance is detrimental for the application since it means that a large quantity of the absorbed sunlight is re-emitted as black-body radiation, and this effect increases as the temperature increases. Conversely, S1, S2, and S3 present values of emittance lower than the untreated sample. The obtained results could be explained as a combination of effects which are due to the surface composition (oxide phases found on the microstructures covering the LIPSS) and microstructures features. For the latter, as reported in Ref. [22], the combined *α* increase and *ε* decrease, or, conversely, the joint *α* and *ε* increase are also connected to the various peculiar sizes and densities of microstructures induced by the laser source impinging onto the molybdenum surface. In particular, the large size of the microstructures found in S4, and their covering by oxidized phases, can explain the highest emittance shown by this sample.

### 3.2. Characterization of the Samples after Thermal Ageing

#### 3.2.1. Morphological Measurements

As reported in the introductory section, one of the aims of this work is to evaluate the morphological, structural, and optical properties shown by Mo LIPSSs after an ageing process designed to reproduce the condition under which the absorber would normally work within a thermionic converter in terms of temperature and pressure. For this purpose, the fabricated sample underwent six thermal cycles from RT to 1100 K, with a stationary state at 1100 K of a duration of approximately 8 h. The ageing process was carried out in high vacuum conditions (base pressure < 1 × 10^−5^ Pa). The sample temperature was measured using a K-type thermocouple mounted on the heating stage on which the samples were placed, as well as using an optical infrared pyrometer (model Minolta Cyclops 152, Tokyo, Japan) monitoring the sample through a quartz viewport as an auxiliary method.

**Figure 10:**
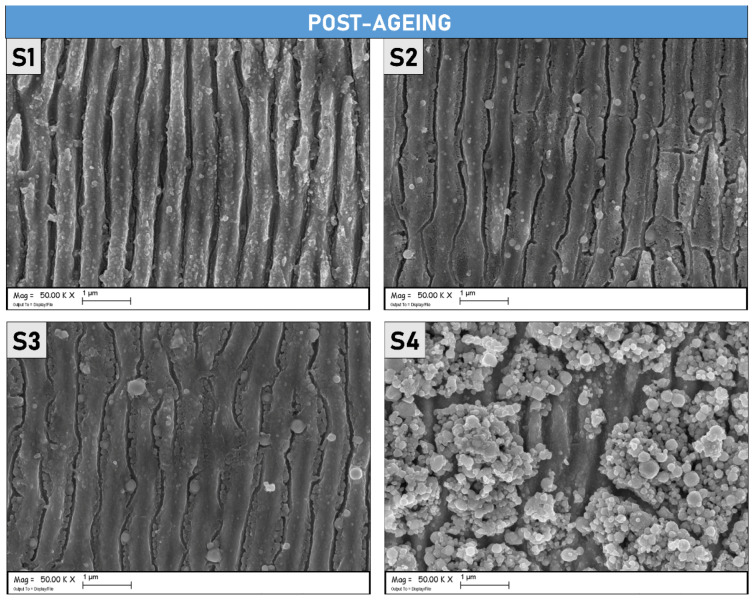
SEM micrographs (magnification 50 k×) of the four different Mo samples after thermal ageing.

**Figure 11:**
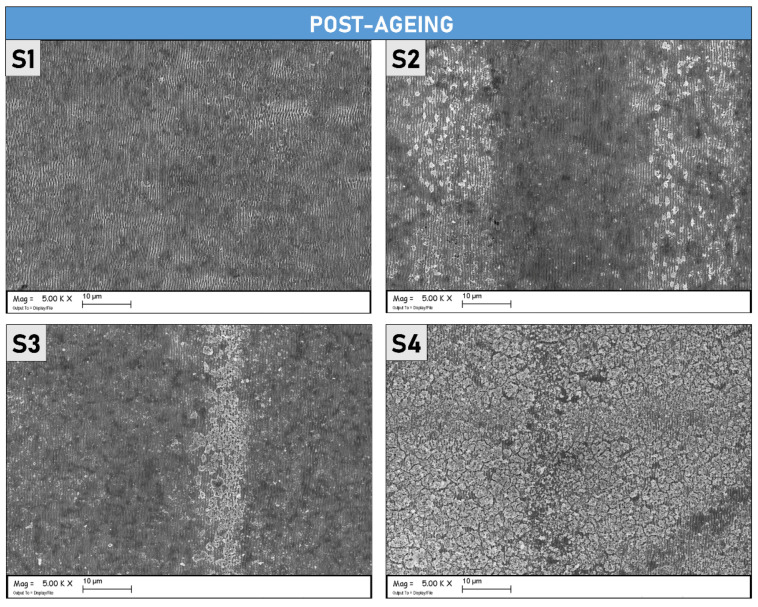
SEM micrographs acquired at lower magnification than Figure 10 (5 k×) of the four different Mo samples after thermal ageing.

The SEM micrographs presented in Figure 10, show the samples after completing the ageing process. As it is possible to observe, the sample S1 does not display notable morphological differences if compared with the images taken before the thermal ageing process (reported in Figure 1). LIPSS on the S1 surface is regular and uniformly distributed, as also demonstrated by Figure 11 which shows SEM micrographs at a lower magnification. It is possible to affirm that the ageing process did not modify S1 on the morphological level and that the regular periodicity was preserved. On the other hand, S2 and S3, present visible cracks between the peaks/ridges of the ripples, suggesting that thermal stress, induced by the ageing process, had an impact on their morphological features. Different, from the S1 sample, the presence of a larger quantity of microstructures due to the treatments, as shown in Figure 1 for S2 and S3, can induce more significant thermal damage caused by structural deformations at the operating temperature. Lastly, S4 is completely covered by randomly distributed microstructures, as can be appreciated in Figure 11. Note that S2 and S3 also present randomly distributed microstructures but are only localized across two contiguous lines of the raster scan.

#### 3.2.2. Optical Measurements

**Figure 12:**
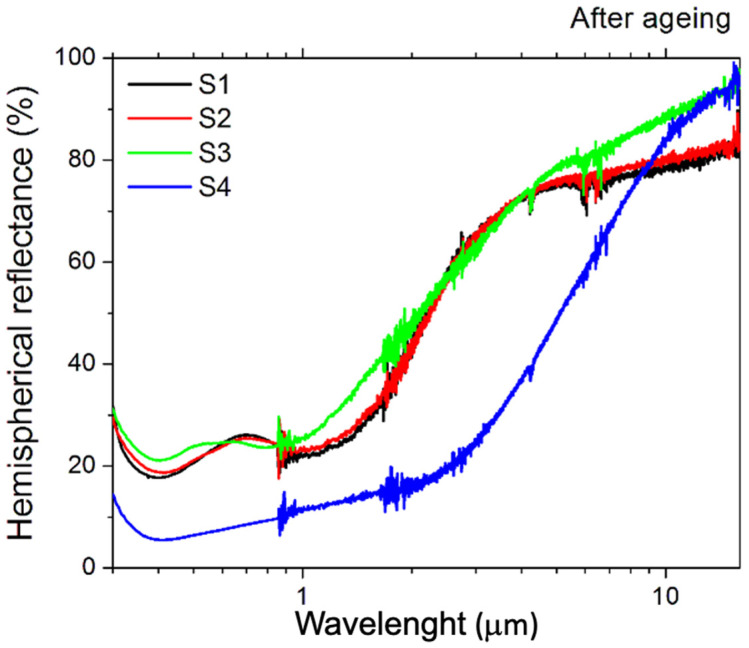
Hemispherical reflectance of the four treated samples after the ageing process.

**Figure 13:**
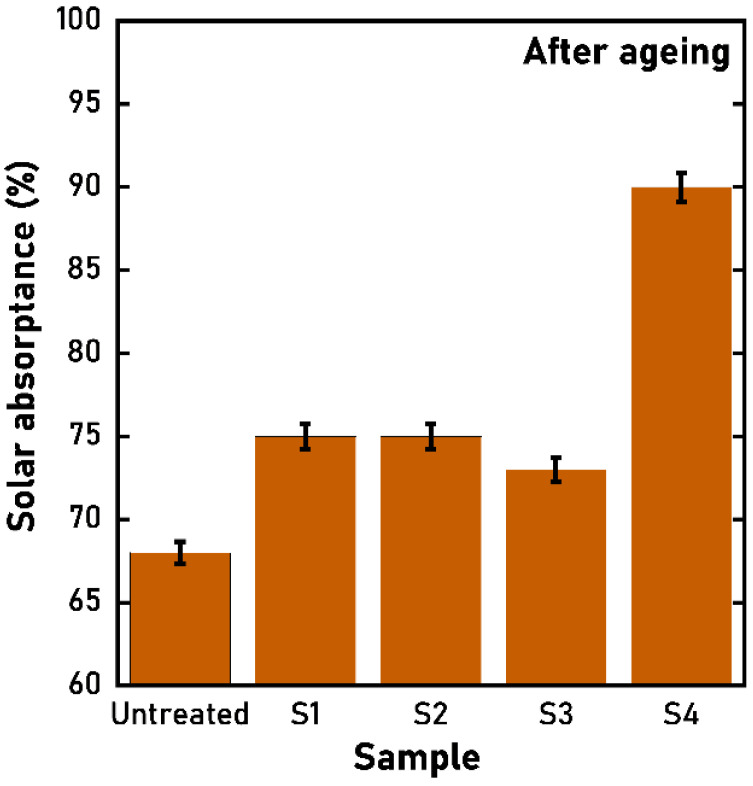
Solar absorptance of the four treated samples and the reference untreated one after the ageing process.

The hemispherical optical reflectance of the aged samples is reported in Figure 12. It is notable that the spectra have similar shapes, with S1, S2, and S3 having lower values than the S4 sample. This is confirmed by the derived solar absorptance, which reveals lower values than the as-treated samples but still higher than the pristine one (Figure 13). S1, S2, and S3 samples have similar solar absorptance values, conversely, the S4 solar absorptance is far higher, achieving a value of 92% (Figure 13).

**Figure 14:**
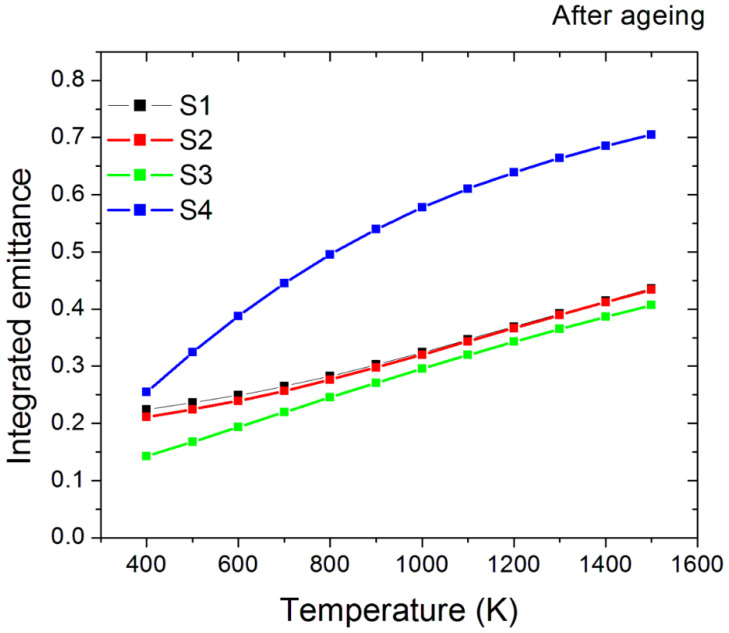
Integrated emittance of the four treated samples and the reference untreated one after the thermal ageing process.

Similar differences are found in the emittance trend as a function of the operating temperature (Figure 14). The emittance of the S1, S2, and S3 samples is still comparable in terms of absolute value and curve shape, with S3 having the lowest values, whereas the S4 sample has a higher emittance.

#### 3.2.3. Raman Spectroscopy

**Figure 15:**
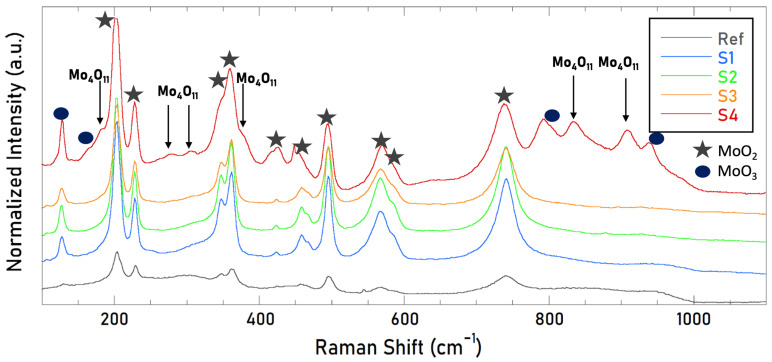
Raman spectra of the four nanotextured samples after the thermal annealing. Bands related to the Mo_4_O_11_ specie are highlighted for the S4 spectra.

Figure 15 shows the Raman spectra of the four nanotextured samples in the wavenumber range 100–1100 cm^−1^, as well as the spectra measured for the reference untreated one (gray line). In all spectra has been observed the presence of MoO_3_ species (120, 157, 816, and 940 cm^−1^) in a prevailing MoO_2_ matrix with Raman bands at 203, 228, 347, 360, 421, 458, 495, 567, 586, 740 ${\mathrm{cm}}^{-1}$, in perfect agreement with the literature [30,31]. We observe that only for the S4 sample the Mo_4_O_11_ specie, with Raman bands at 182, 272, 311, 378, 834, and 910 ${\mathrm{cm}}^{-1}$, was identified.

**Figure 16:**
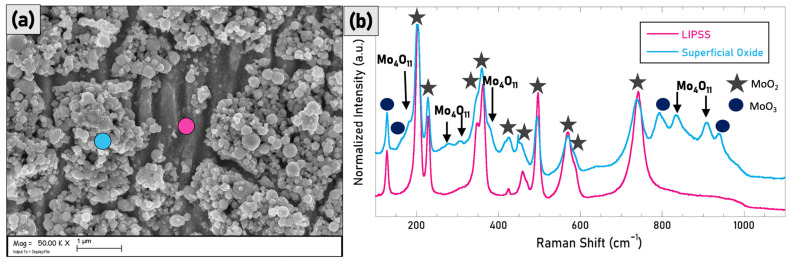
In (**a**), the SEM image of the S4 sample in which the difference between the underneath LIPSSs (pink point) and the upper nanostructures (blue point) is clearly visible. In (**b**), Raman spectra have been acquired at the same points indicated in the micrograph.

The presence of Mo_4_O_11_ only in S4 has been further investigated. Matching the information obtained by SEM and Raman spectroscopy is possible to see in Figure 16 that the deposited microstructures are mainly the Mo_4_O_11_ species (blue spectrum) on the rastered prevailing MoO_2_ surface (pink spectrum).

## 4. Discussion

### 4.1. Solar Selectivity and Thermal Stability

The main parameter for evaluating the performance of a solar absorber is the spectral selectivity, also called the *α*/*ε* ratio. Ideally, a solar absorber should exhibit a low emittance to avoid thermal radiation losses; therefore, spectral selectivity *α*/*ε* should be as high as possible (i.e., close to 1).

**Figure 17:**
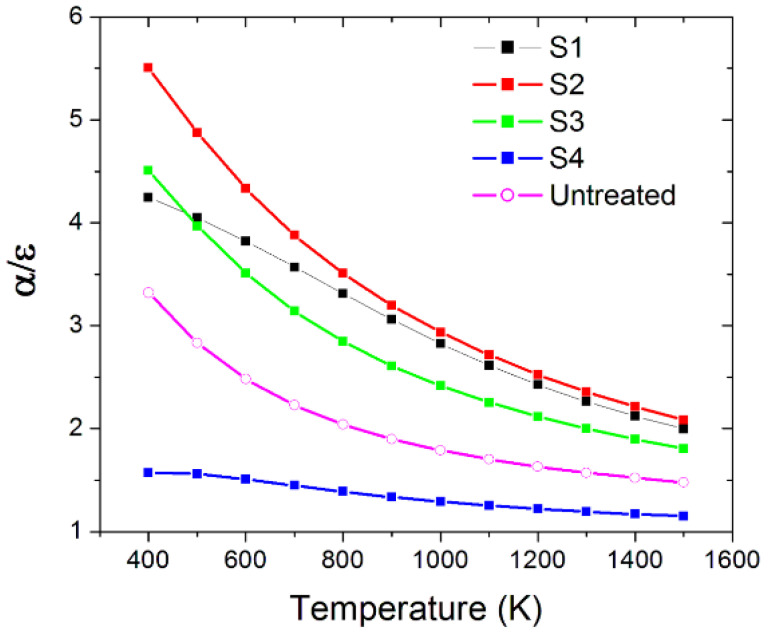
Spectral selectivity (*α*/*ε* ratio) of the four nanotextured samples compared to the untreated material as a function of the temperature before the thermal annealing process.

Figure 17 shows *α*/*ε* in the temperature range of 400–1500 K for the samples before the thermal annealing. Considering T = 1100 K as the operating temperature of practical applications, we note that laser texturing increases spectral selectivity up to values of approximately 2.7 for the best sample S2, more than 1.5 times higher than the untreated sample. These results are extremely promising considering that the most advanced solutions developed and specifically optimized for acting as solar absorbers present a value α/ε > 2.5, but with a much more complex structure than in our case [5]. On the other side, it is important to notice that when the laser fluence is lower than 10 J/cm^2^ (samples S1, S2, and S3) the performance is very similar, even though S1 shows the most effective morphology (in terms of both depth and order of the nanostructures) among the differently treated samples.

**Figure 18:**
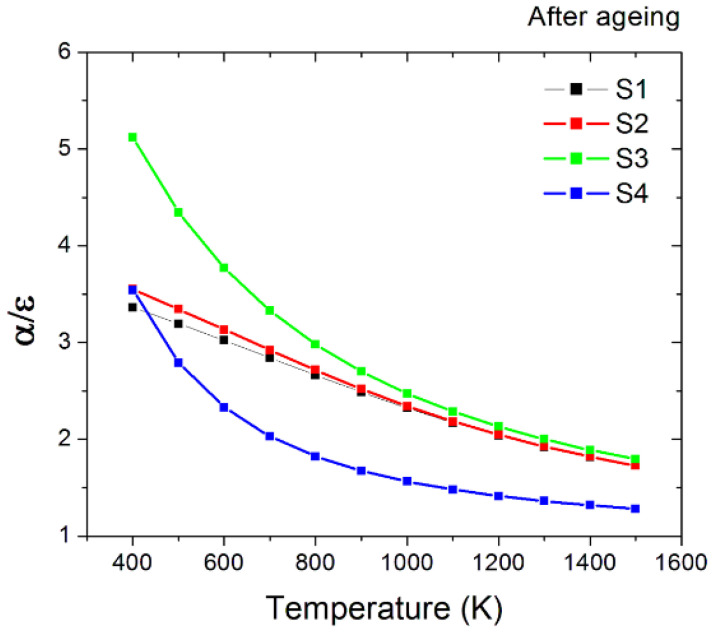
Spectral selectivity (*α*/*ε* ratio) of the four nanotextured samples compared to the untreated material in the function of temperature after the thermal annealing process.

After the ageing process, S1 and S2 samples show a reduction of the α/ε values, while S3 and S4 show an increase, more significant at lower temperatures (Figure 18). Solar absorptance is lower for all samples after ageing, but emittance is lowered for S3 and S4 and slightly raised for S1 and S2. Since the morphology of the surfaces appears to be unaltered for the S1 sample, it seems that there is also a different factor to reduce the performance beyond the structural dislocations (identified for samples S2 and S3). On the other hand, the analysis of the Raman data does not indicate any clear difference in the surface chemistry of the samples. Finally, we can state that the performance superiority of the S3 sample depends on its emittance which is the lowest one among all the samples.

**Figure 19:**
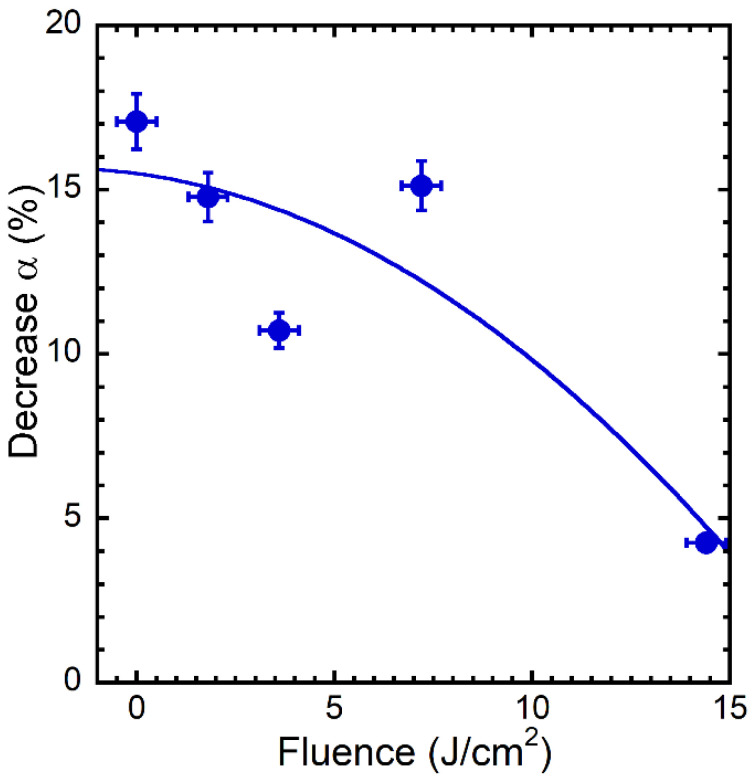
Decrease (expressed in percentage) of the solar absorptance α for the different samples after the thermal annealing with respect to the not annealed α values measured as a function of the laser fluence.

Interestingly, the S4 sample presents the less significant reduction of solar absorptance, lower than 5% as indicated in Figure 19. Conversely to the other samples, S4 shows an improvement of the performance after the ageing for all the reported range of temperatures, despite remaining the worst sample considering the absolute values for T > 500 K. This behaviour can be surely associated with the presence of the Mo_4_O_11_ nanoparticles formed on the top of the LIPSSs, which act as coverage of the nanostructured surface by avoiding further modifications of the structures.

The overall analysis of SEM images, Raman spectroscopy data, and optical properties indicated that the light trapping induced by the laser treatments is the most effective effect for improving the performance of solar absorbers, in agreement with previous findings on tantalum diboride [22] with better performance than the untreated Mo plate when the laser fluence is lower than 10 J/cm^2^ but without a further improvement due to a higher degree of structural homogeneity. The formation of Mo_4_O_11_ nanoparticles which occur at fluences higher than 10 J/cm^2^ must be deeply investigated in the next future thanks to the beneficial effect on the preservation of the optical properties during operations.

### 4.2. Formation of Nanostructured Mo Oxides and Their Role: Future Challenge

In this work, we can mainly identify two different regimes for the ultra-short pulse laser treatments: when the fluence is lower than 10 J/cm^2^, LIPSSs are obtained with a fixed periodicity and few structural defects; when the fluence is higher than 10 J/cm^2^, the formation of oxide, nanostructured after long thermal annealing, occurs covering the ripples present onto the material’s surface.

In the past, nanostructuring molybdenum thin films by an ultrashort pulsed laser source (λ = 1064 nm; τ = 290 fs and *f* = 78.5 MHz) with power varying in the range of 0–1 W has shown that different oxide compositions MoO_x_ could be generated onto the material’s surface. In detail, the early native MoO_3_ thin layer present on the molybdenum surface can be replaced by intermediate MoO_3-x_ oxides due to the generation of oxygen vacancies type defects progressively generated by increasing the laser power up to 1 W when, mainly, the MoO_2_ is detected. These oxides thin layers (10–100 nm) can display selective high UV-Vis-NIR absorbance due to the average reflectance ≤5% revealed in the range 190–1100 nm and, therefore, meet the requirements of selective solar absorbance devices [32]. Recently, Liu et al. [33] have demonstrated that by varying the scanning interval during LIPSS performed in the air the deposition of amorphous nanoparticles together with the hierarchical surface nanostructure can be provided. While the hierarchical surface nanostructure is due to the LIPSS, the nanoparticles are generated by recondensation and oxidation of plasma’s gaseous species (metal’s atoms/clusters/ions) produced by the laser ablation (LA) process induced by the incident laser beam and reacting with the excited and reactive species of the surrounding atmospheric environment. Scanning intervals in the range of 1-20 mm together with an ultrashort pulsed laser source (λ = 1030 nm; τ = 400 fs; *f* = 400 kHz; laser power = 5 W) were used. Different LA nanoparticles densities have been deposited onto the underlying LIPSS nanostructures by varying the scanning range interval with the 1 µm and 20 µm showing the highest and lowest density, respectively. For the former microporous aggregates could be revealed covering the LSFL with spatial period ⋀ in the range of 600–800 nm. In these circumstances the decrease of selective UV-VIS-NIR-MIR absorbance spectra was detected by diminishing the nanoparticles density in turn obtained by gradual increase of the scanning intervals. This outcome shows that varying the process parameters, the combination of LA and LIPSS effects can be tuned for adjusting the optical material’s properties. The observed low reflectance observed was related to light trapping due to hierarchical LIPSS nanostructures and absorption of the deposited nanoparticles as well as light trapping played by surface dispersion/aggregation of these. Reflectance spectra have evidenced that selective absorbances of 96–99% and 50–86% can be reached in the UV-Vis-NIR and NIR-to-MIR spectral range, respectively. About NIR-to-MIR absorbances sub-stoichiometric MoO_x_ nanomaterials play a relevant role due to localized surface plasmon resonances induced by oxygen vacancies.

It follows that a complex regime of processes was induced by the laser fluences used in the present work where LIPSSs ripples as well as variable compositions can be formed, such as Mo_4_O_11_ and MoO_2_. A further investigation could be considered in advance for tuning the LIPSSs treatments for specific opto-electronic applications, considering that the electronic band structures of these oxides span from a semiconductor behaviour with a wide bandgap energy of approximately 3 eV for MoO_3_ (the most diffused Mo oxidized state) to a semi-metallic character of MoO_2_ due to the presence of Mo 4d bands below its Fermi energy level, whereas for Mo_4_O_11_ a quasi-metallic behaviour can be assessed [34,35].

Finally, it is important to underline that, conversely to the method applied in this work, the formation of MoO_2_ and Mo_4_O_11_ is more difficult to obtain using a more conventional temperature and gas environment: for instance, MoO_3_ can be reduced to orthorhombic Mo_4_O_11_ in presence of hydrogen and temperatures above 425 °C which, after consecutive reactions, is reduced into MoO_2_ [36], or MoO_3_ nanomaterials can be reduced at about 700 °C in a N_2_ flow reactive plasma where a combination of MoO_2_ and Mo_4_O_11_ phases are determined [37].

## 5. Conclusions

Surface treatments using an fs-laser source with different fluences in the range of 1.8-14.4 J/cm^2^ have been successfully performed on commercial Mo plates. A periodicity of 550 ± 60 nm has been assessed for the formed LIPSSs, with a variable depth in the range of 100–300 nm as a function of the laser fluence. The presence of Mo oxides has been detected in all the samples, with a more significant quantity in the sample treated at the highest fluence (S4, 14.4 J/cm^2^) where stable phase suboxide Mo_4_O_11_ microstructures have been determined by µ-Raman spectroscopy. The treatments have improved the performance of Mo plates as solar absorbers, with a maximum selectivity α/ε of about four times occurring at 800 K for the best treated sample. The ageing process, consisting of long thermal annealing, evidenced, for the first time to our knowledge for these molybdenum laser-induced nanostructures, that the surface chemistry changes, with a probable modification of the oxide species, even if the selectivity remains almost unaltered. Interestingly, the sample S4 showing a structural modification of the oxides after the annealing, with the formation of the nanostructured species Mo_4_O_11_, opens a new route for employing laser treatments of molybdenum even for its application in either photoelectrochemical or sensing devices.

## Figures and Tables

**Table 1:** Experimental parameters of the four different nanotexturing treatments.

Sample	Single Pulse Fluence (J/cm^2^)	Longitudinal Translational Speed (10^−3^ m/s)	Number of Pulses per Spot
S1	1.8	2.0	30
S2	3.6	2.0	30
S3	7.2	2.0	30
S4	14.4	2.0	30

## Data Availability

Not applicable.
